# From rib fixation to dynamic chest wall reconstruction: historical lessons for modern surgical stabilization of rib fractures

**DOI:** 10.1007/s00068-026-03324-z

**Published:** 2026-09-04

**Authors:** David Sutori, Thomas W. White, Zachary M. Bauman, SarahAnn S. Whitbeck, Christopher Spering

**Affiliations:** 1 Thoracic Surgery Unit, Department of Surgery, Aladar Petz University Teaching Hospital, Gyor, Hungary; 2 Doctoral School, University of Pecs, Pecs, Hungary; 3 Division of Trauma and Acute Care Surgery, Intermountain Medical Center, Murray, USA; 4 Department of Surgery, University of Nebraska Medical Center, Omaha, USA; 5 Chest Wall Injury Society, Salt Lake City, USA; 6 Department of Trauma Surgery, Orthopaedics and Plastic Surgery, Goettingen University Medical Center, Universitaetsmedizin Goettingen, Robert-Koch-Strasse 40, 37075 Goettingen, Germany

**Keywords:** Surgical stabilization of rib fractures, SSRF, Rib fractures, Flail chest, Chest wall stabilization, Thoracic trauma, Intramedullary fixation, Historical review

## Abstract

**Purpose:**

Surgical stabilization of rib fractures (SSRF) has re-emerged as an important component of chest wall trauma care, yet its history is often presented as a simple timeline rather than as a source of operative and biomechanical lessons. This literature, problem-oriented historical review re-examines earlier rib fixation strategies in order to identify the mechanical and operative problems they were intended to solve, why many disappeared, and which principles remain relevant to contemporary SSRF practice.

**Methods:**

A literature review was conducted focusing on historically important fixation techniques and conceptual milestones in the management of serious rib fractures and flail chest. PubMed/MEDLINE and Google Scholar were searched from database inception to 19 March 2026 using terms related to rib fracture fixation, flail chest, SSRF, intramedullary fixation, and history. Studies were purposively selected if they described key fixation concepts, major shifts in management strategy, recurrent technical problems still seen in modern SSRF, or contemporary guideline context. Forty-seven articles were included and synthesized using a problem-based rather than purely chronological framework.

**Results:**

Across eras, rib fixation evolved around recurring challenges: (1) restoration of chest wall stability and thoracic continuity, (2) the tension between internal pneumatic stabilization by positive-pressure ventilation and structural repair, (3) difficult access to posterior, subscapular, and upper-rib fractures, (4) implant–rib mismatch and preservation of the intercostal neurovascular bundle, and (5) a limited evidence base. Historical methods - including suture and wire cerclage, clamp-based plates, intramedullary devices, and percutaneous or limited-incision techniques - were early attempts to solve these problems rather than obsolete curiosities. Their main limitations were invasive exposure, bulky and excessively rigid implants, poor conformity to rib curvature, neurovascular irritation, and weak clinical evidence. The most important transition was conceptual: reframing rib fracture patterns as dynamic chest wall instability in which a static CT fracture map must be translated into an operative construct that restores functional thoracic mechanics while preserving the muscular and soft-tissue envelope.

**Conclusions:**

Modern SSRF represents the maturation - not the replacement - of historical fixation concepts under improved biomechanical, biological, and evidentiary conditions. The lasting lessons are that fixation should target the mechanically relevant instability, balance stability with biological restraint, match access strategy to fracture location and chest wall anatomy, and expand indications selectively and discipline. Reinterpreting historical techniques through this problem-oriented lens may help refine current SSRF practice, avoid repetition of past errors with new implants, and guide future development of dynamic chest wall reconstruction.

**Supplementary Information:**

The online version contains supplementary material available at https://doi.org/10.1007/s00068-026-03324-z.

## Introduction

Thoracic trauma remains a major contributor to trauma-related morbidity and mortality, and rib fractures are the most common osseous injury of the chest. Although non-operative treatment remains the cornerstone for many patients, surgical stabilization of rib fractures (SSRF) has gained increasing acceptance over the last two decades as evidence has accumulated for selected injury patterns and patient subgroups. At the same time, rib fracture populations remain heterogeneous, and contemporary management is still characterized by variation in triage, patient selection, timing, operative strategy, nonoperative care, and implant choice [1–3]. 

The modern surgency of SSRF has been driven first by data in flail chest and, more recently, by studies in selected non-flail fracture patterns. Landmark prospective studies demonstrated that operative fixation in severe flail chest can reduce ventilator dependence, pneumonia, and intensive care utilization compared with internal pneumatic stabilization or best medical management [4, 5]. Subsequent prospective and multicenter clinical trials extended this discussion beyond classic flail chest, suggesting that selected patients with multiple displaced non-flail rib fractures may benefit in terms of pain, respiratory outcomes, and pleural-space complications [6, 7]. Nevertheless, even with growing evidence and broader guideline support, major questions remain under active debate, including exactly who benefits most, when fixation should be performed, how exposure should be minimized, which constructs best balance stability with respect for chest wall biology, and long terms patient reported outcomes [1, 2]. 

Importantly, SSRF is not a modern invention. Rib fracture stabilization was described as early as the beginning of the twentieth century, when Scudder reported primary suture fixation for fractured ribs, and multiple subsequent concepts later emerged, including wire cerclage, intramedullary support, percutaneous suspension methods, clamp-based plates, limited-incision techniques, and titanium systems designed to better conform to rib anatomy [8–10]. However, previous historical reviews have been primarily chronological and descriptive. They have clarified who introduced major techniques and when key transitions occurred, but they have not fully addressed a different and clinically more relevant question: which mechanical and operative problems these older techniques attempted to solve, why many of them fell out of favor, and which principles survived into modern chest wall stabilization. The aim of the present review is therefore not merely to recount the history of SSRF, but to examine forgotten or abandoned fixation concepts through a problem-oriented lens and to identify the lasting lessons they offer for current SSRF indications, approach selection, implant design, and future development. Unlike prior timeline-based historical overviews, this review provides a clinically actionable framework by linking abandoned fixation strategies to current debates on indication discipline, surgical exposure, implant design, and the selective expansion of SSRF beyond classic flail chest. This interpretation also changes how the modern problem is framed. Contemporary chest wall surgery is not just the technical ability to fix ribs. The injured thorax has to be read as a moving biomechanical system. In flail chest or severe displacement, CT defines the fracture anatomy, but it does not, by itself, explain how the segment will behave during breathing, coughing, pain-limited ventilation, or progressive displacement. Modern SSRF has evolved around that translation: from a static fracture map to an operative plan that restores mechanics while limiting injury to the muscular and soft-tissue envelope [1, 11–18]. 

Previous historical reviews by Bemelman et al. and Shaban et al. have provided valuable chronological overviews of major fixation techniques, clarifying when key methods were introduced and when they fell out of use [8, 9]. However, these timeline-based accounts offer limited guidance on how earlier concepts should inform present-day decisions about indications, access strategy, implant design, and construct planning. The present review differs in three important ways. First, it is structured around recurring clinical and mechanical problems—chest wall instability, the trade-off between internal pneumatic stabilization and structural repair, access to posterior and subscapular fractures, implant–rib mismatch, and evidentiary weakness—rather than around dates and devices. Second, it explicitly links historical solutions and failure modes to contemporary SSRF debates, including WSES/CWIS guidance, randomized and prospective data in flail and non-flail patterns, and the emergence of rib-specific titanium systems and newer biomaterials [1, 2, 4–7, 14, 19]. Third, it reframes the injured thorax as a dynamic biomechanical system and derives practical lessons for construct completeness, muscle-sparing exposure, and phenotype-specific indications. In this way, the history of rib fixation is used not merely to catalogue obsolete implants, but to support a clinically actionable framework for modern dynamic chest wall reconstruction.

### Review approach

This review was designed as a literature, problem-oriented historical review rather than a systematic review or meta-analysis. Because the objective was interpretive historical synthesis rather than exhaustive evidence retrieval, PRISMA-based reporting was not applied. Instead, the review was structured and reported in a transparent manner, with attention to key quality domains emphasized by the SANRA scale for literature review articles, including the relevance and aims of the review, literature search description, referencing, presentation of evidence level, and discussion of clinically relevant endpoints. The literature search was conducted on the 19th of March, 2026 in PubMed/MEDLINE and Google Scholar, with additional hand-searching of reference lists from key historical reviews, technical papers, clinical studies, and contemporary guidance documents. No formal lower date limit was applied because the aim of the review was to capture the historical development of rib fixation from its earliest descriptions to current SSRF practice; the search covered the period from database inception to the 19th of March 2026.

Search terms included combinations of “rib fracture fixation,” “flail chest fixation,” “surgical stabilization of rib fractures,” “SSRF,” “rib plating,” “cerclage,” “Judet,” “intramedullary rib fixation,” “thoracic trauma,” “chest wall stabilization,” “history,” and “historical.” Because this was a literature, problem-oriented historical review rather than a systematic review, the search was not intended to be exhaustive, and the included studies represent a purposive sample selected for conceptual relevance rather than a comprehensive evidence base.” Instead, studies were purposively selected if they fulfilled at least one of the following criteria: (1) description of a historically important fixation technique or operative principle; (2) representation of a major conceptual shift in the management of rib fractures or flail chest; (3) illustration of a recurring technical problem that remains relevant in modern SSRF; or (4) provision of contemporary clinical or guideline context necessary to interpret historical developments from a present-day perspective. Preference was given to publications that either introduced a key fixation concept, clarified why a technique failed or persisted, or informed current debates on indications, access, implant design, and selective expansion of SSRF. After screening and conceptual selection, 47 articles were included in the final synthesis and are summarized in the supplementary table.

The final body of literature was synthesized using a problem-based rather than purely chronological framework. For each major historical technique, the analysis focused on four questions: what clinical or mechanical problem the technique attempted to solve, why it was adopted, why it disappeared or remained marginal, and which underlying principle persisted into contemporary SSRF practice. As a literature historical review, this work does not claim completeness and remains subject to selection and interpretation bias; however, the explicit search strategy and inclusion framework were intended to improve transparency and reproducibility.

### Historical problems that shaped chest wall stabilization

Rib fixation evolved around a recurring practical problem: how to control an unstable chest wall. Severe rib fractures and flail chest are visible as osseous injuries on imaging, but their clinical relevance comes from motion—paradoxical movement, muscle pull, pain, displacement, impaired cough, and loss of thoracic continuity. The historical challenge was not simply to put hardware on ribs. It was to restore useful chest wall mechanics without creating a second injury through the exposure [1, 8, 9, 11–13, 20]. Early surgeons recognized this problem clinically, even before modern imaging and rib-specific implants were available. Their primary challenge was to restore chest wall stability and re-establish thoracic continuity [8, 9]. 

A second major problem was how that stability should be achieved. Historically, treatment strategies were divided between internal support and external support [8, 9]. Internal pneumatic stabilization by positive-pressure ventilation eventually became dominant, largely because many early fixation methods were invasive, technically limited, and supported only by weak evidence [8, 9]. This long-standing tension between physiological support and structural restoration remains central to modern SSRF thinking [1, 8, 9]. 

Surgical access and exposure was another persistent issue. Posterior, subscapular, and upper rib fractures have always been difficult to expose, which explains the historical interest in percutaneous, suspension-based, and intramedullary solutions [8, 9, 11, 20]. Modern muscle-sparing, minimally invasive, and thoracoscopic-assisted SSRF approaches reflect the same underlying problem: how to achieve fixation without excessive exposure-related morbidity [11, 21–23]. 

Rib fixation was also shaped by implant–rib mismatch and the need to preserve surrounding structures [8, 9, 11, 12, 20]. Unlike other long bones, ribs are thin, curved, elastic, and continuously mobile. Earlier systems often struggled with poor conformity, excessive rigidity, loosening, or neurovascular irritation [8, 9, 11, 12, 20]. For that reason, many historical techniques are best understood not as failed curiosities, but as early attempts to solve problems that still define contemporary SSRF: stability, safe exposure, implant design, and biological tolerance. The final historical obstacle was evidentiary weakness; most older techniques were described in small series or technical reports, which limited durable adoption until modern implants and stronger clinical data influenced the field of chest wall trauma [2, 4, 5, 8, 9]. 

### Forgotten techniques and what they were trying to solve

The earliest operative fixation methods, beginning with suture repair and later wire cerclage, were direct attempts to restore continuity in markedly displaced or comminuted rib fractures before dedicated chest wall implants existed [8–10]. Their appeal was technical simplicity and immediate mechanical reduction, but their limitations were lack of construct standardization, variable durability, and limited reproducibility [8, 9, 20]. Even so, these methods established the core principle that chest wall instability could be treated by mechanical restoration rather than supportive care alone [8, 9, 20]. 

Clamp-based and early plate systems were introduced to improve fixation strength and reduce some of the shortcomings of cerclage [8, 9, 20, 24]. Early modern plate-and-screw osteosynthesis represented a transitional step between historical clamp-based constructs and contemporary rib-specific fixation systems. Engel et al. described operative stabilization of flail chest using osteosynthesis plates, including pelvic, mandibular, and customized reconstruction plates, thereby illustrating both the renewed interest in rigid chest wall reconstruction and the lack of consensus on optimal implant design before dedicated rib-specific systems became widely adopted [25]. Judet-type devices and related constructs were attractive because they aimed to stabilize the rib without relying entirely on circumferential fixation and, in some designs, avoided trans-rib drilling [8, 9, 20, 24]. However, poor conformity to rib curvature, implant bulk, excessive rigidity, and the risk of intercostal neurovascular irritation limited widespread adoption [8, 9, 20, 24]. These systems are historically important because they framed a problem that remains central to modern SSRF: how to achieve sufficient stability without paying for it with hardware pain, soft-tissue injury, or biologic mismatch [8, 9, 11, 20, 24]. 

Intramedullary fixation concepts emerged largely as a solution to access rather than purely to biomechanics. Posterior, subscapular, and otherwise difficult-to-expose fractures have always challenged open plate fixation, and intramedullary devices offered a way to stabilize these injuries through less extensive dissection [8, 9, 11, 20, 26]. Historical systems were imperfect and vulnerable to migration or fixation failure, but the concept survived because it addressed a real anatomical problem [8, 9, 26]. Modern series and technical analyses continue to support intramedullary fixation as a useful option in selected posterior fractures or fractures beneath the scapula, especially when conventional plating would require disproportionately morbid exposure [11, 20, 26]. 

Percutaneous and limited-incision techniques were driven by the same concern from another angle: exposure morbidity. Historical reports of self-made plates and less invasive insertion strategies show that the ambition to reduce operative trauma long predates modern Minimally Invasive Plate Osteosynthesis (MIPO) and thoracoscopic-assisted SSRF [8, 9, 21–23]. What prevented these techniques from becoming dominant was not lack of conceptual value, but limitations in materials, implant design, instrumentation, imaging, and evidence quality [8, 9, 11, 20]. This helps explain why many early methods should not be viewed as failed curiosities, but as early responses to enduring problems—stability, access, implant–rib compatibility, and tissue preservation—that modern SSRF continues to refine rather than replace [8, 9, 11, 20]. 

SSRF continued to evolve as older ideas became technically usable. Better implants, imaging, perioperative care, operative standardization, and clinical data allowed surgeons to advance mechanical restoration under safer and more reproducible conditions. Randomized and prospective studies in flail chest, followed by prospective data in selected non-flail patterns, gave the field the clinical legitimacy that earlier techniques had lacked [4–6]. The principle had not disappeared; the means to apply it safely, reproducibly, and with convincing outcome data had been missing [1, 2, 8, 9, 11, 20]. 

Table 1 distills the central argument of this review: modern SSRF did not emerge from entirely new principles, but from the refinement of older fixation concepts under better biomechanical, biological, and evidentiary conditions.

**Table 1:** Historical rib fixation concepts, their limitations, and lessons for modern SSRF This table summarizes the central argument of the review: modern SSRF did not emerge from entirely new principles, but from the refinement of older fixation concepts under better biomechanical, biological, and evidentiary conditions

Historical concept	Main problem addressed	Why it was attractive at the time	Main limitation(s)	What survived into modern SSRF	Current practical implication
Static fracture-based planning	Identification of visible osseous discontinuity and flail segments	CT and radiography allowed fracture mapping and operative targeting	Static imaging alone may underestimate dynamic instability, muscular envelope dysfunction, progressive displacement, and residual motion in incompletely stabilized flail patterns	The need to translate fracture anatomy into a functional chest wall construct	SSRF planning should identify the mechanically relevant instability, not only the most visible fractures; construct design should restore dynamic thoracic mechanics while preserving muscle and soft tissue function
Suture fixation / wire cerclage	Restoration of chest wall continuity in displaced or comminuted rib fractures before dedicated implants existed	Technically simple, immediately available, and capable of direct mechanical reduction	Limited standardization, variable durability, construct instability, and soft-tissue burden from circumferential fixation	The core principle that selected rib fractures may require **mechanical restoration**, not supportive care alone	SSRF should still be understood primarily as restoration of chest wall mechanics rather than as hardware placement alone
Clamp-based plates (e.g. Judet-type concepts)	Need for stronger fixation than cerclage, often while reducing reliance on full circumferential fixation	Offered more rigid stabilization and more controlled reduction than simple suture or wire techniques	Implant bulk, poor conformity to rib curvature, excessive rigidity, hardware pain, intercostal neurovascular irritation, and limited biological tolerance	The importance of **stable fixation with minimal biological cost** and of rib-conforming implants	Modern implant selection should prioritize rib-specific, lower-profile constructs that provide sufficient stability without excessive rigidity, implant bulk, or neurovascular irritation
Intramedullary fixation concepts	Difficult access to posterior, subscapular, or otherwise hard-to-expose fractures	Allowed stabilization through less extensive exposure when formal plating required broader dissection	Migration, cut-out, loss of fixation, limited rotational control, and reduced suitability for complex segmental patterns	The principle that **access should influence implant choice**	Selected posterior or subscapular fractures may still be better served by intramedullary or hybrid strategies when open plating would be disproportionately morbid
Percutaneous / limited-incision techniques	Reduction of exposure-related morbidity	Attempted to achieve fixation while limiting operative trauma and soft-tissue injury	Crude instrumentation, limited imaging, poor implant design, weak evidence base	The enduring goal of **minimizing exposure-related biological cost**	The modern lesson is muscle-sparing exposure rather than the shortest possible incision; thoracoscopic-assisted and MIPO strategies are refinements of an old need, not ends in themselves
Early rigid plate constructs / non-rib-specific plate-and-screw osteosynthesis	Need for stronger mechanical stabilization of unstable chest wall injuries	Improved initial fracture control compared with simpler fixation methods	Poor implant-rib match, excessive stiffness, pain, loosening, hardware intolerance, and reduced tolerance in a thin, curved, mobile thoracic cage	Recognition that ribs are **not long bones** and require rib-specific fixation strategies	Implant selection must follow chest wall biology, curvature, and respiratory motion rather than long-bone fixation logic
Segmental / flail fixation strategies with incomplete stabilization	Need to control mechanically relevant instability in multilevel or flail patterns	Technically easier and less extensive than fixing every clinically important fracture	Residual motion at non-plated fractures, continued deformity or displacement, and increased stress on the construct	The principle that **construct adequacy depends on what is fixed**,** not simply on whether hardware is placed**	In flail or segmental patterns, fixation should neutralize the clinically important instability and may require fixation of both fractures in a segmental rib rather than partial plating alone

### Lasting lessons for modern SSRF

SSRF is best understood as treatment of chest wall instability, not as fixation of isolated rib fractures. The operation succeeds when the construct controls the part of the thorax that is actually unstable. In practical terms, the surgeon has to convert the CT-based fracture map into a dynamic plan: which segments move, which ribs carry the deforming forces, which muscular attachments must be preserved, and which less-displaced fractures may later collapse or deform if they are ignored [1, 8, 9, 11, 12, 14–17, 27]. 

The second enduring lesson is that successful SSRF depends on balancing stability with biological respect. Historical fixation methods repeatedly failed when mechanical control was achieved at the cost of excessive rigidity, poor conformity to rib curvature, implant bulk, or injury to surrounding soft tissues and the intercostal neurovascular bundle [8, 9, 11, 12, 20]. Modern systems are hence not superior because they introduced an entirely new principle, but because they apply the same mechanical objective with improved implant design, lower-profile constructs, rib-specific contouring, and greater respect for chest wall mechanics and soft-tissue preservation [1, 2, 8, 9, 11, 12, 20]. 

Access is not secondary to fixation; it is part of fixation strategy. Posterior, subscapular, and upper-rib fractures have always forced surgeons to choose between exposure and morbidity. Intramedullary, percutaneous, limited-incision, muscle-sparing, and thoracoscopic-assisted methods are different answers to the same problem: stabilize the relevant fracture pattern without creating unnecessary exposure-related damage [11, 20–23, 26, 28]. 

The fourth lesson is that implant choice must follow rib-specific anatomy and mechanics rather than long-bone logic. Ribs are thin, curved in more than one plane, variably mineralized, and continuously loaded by respiration, coughing, muscle pull, and shoulder-girdle motion. In design terms, this translates into several practical requirements: a low-profile implant to reduce prominence and soft-tissue irritation; sufficient contourability to match rib curvature; controlled flexibility rather than excessive rigidity; secure fixation in limited bone stock; and careful respect for the intercostal groove and neurovascular bundle. Historical failures already demonstrated the consequences of implant–rib mismatch. Modern titanium systems addressed many of these problems through rib-specific plate geometry, lower-profile constructs, and improved instrumentation, while newer materials such as PEEK should be understood as attempts to further refine the balance between stability, flexibility, imaging compatibility, biocompatibility, and hardware tolerance [1, 2, 8, 9, 11, 19, 20]. 

### The slow start for SSRF – and its recent rapid gain in popularity

The surgical stabilization of rib fractures, like most new surgical procedures and techniques, got its slow start because earlier fixation methods were invasive, technically inconsistent, poorly standardized, and supported mainly by case reports or small series with limited follow-up. At the same time, positive-pressure ventilation offered a nonoperative form of “internal pneumatic stabilization” that was easier to implement, reproducible, and increasingly compatible with modern critical care [4, 8, 9]. For decades, this combination of technical limitations and the practical appeal of mechanical ventilation pushed operative chest wall stabilization into the background [8, 9]. 

Older fixation systems also suffered from a design problem that modern readers should not underestimate. Many constructs were too bulky, too rigid, poorly matched to rib curvature, or carried a meaningful risk of intercostal neurovascular irritation, hardware pain, loosening, or migration [8, 9, 11, 12, 20]. In other words, historical fixation often failed not because the concept of restoring chest wall continuity was wrong, but because the available materials, operative exposure, and implant geometry were not yet adequate for a thin, curved, mobile structure like the rib cage [8, 9, 11, 12, 20].

The recent exponential utilization of SSRF was therefore not a sudden conceptual revolution, but the convergence of better technology and better evidence. Implant design improved from crude wires and bulky clamp-based devices to lower-profile, more contourable, rib-specific fixation systems. At the same time, modern imaging, perioperative care, regional analgesia, and trauma systems made patient selection and operative planning more reliable. These changes made it possible to better expand an old mechanical principle under conditions that were safer, more reproducible, and technically more defensible [1, 2, 8, 9, 20]. 

Crucially, the modern advancement of SSRF was legitimized by clinical data. Randomized and prospective studies in flail chest showed reductions in pneumonia, ventilator dependence, tracheostomy, and ICU utilization in selected populations treated operatively, and later systematic reviews and meta-analyses largely supported benefit in flail chest, while also emphasizing the limitations of the evidence base and uncertainty around mortality effects [4, 5, 29–32]. Registry-based and contemporary pooled analyses have further suggested that timing and center-level practice patterns may also influence outcomes [33, 34]. These data shifted SSRF from a technically interesting option to a credible part of evidence-based chest trauma care [33, 34].

The next phase of SSRF expansion extended beyond classic flail chest. Prospective data from the CWIS NONFLAIL trial supported benefit in carefully selected patients with multiple severely displaced non-flail rib fractures, suggesting that the value of SSRF may not be confined to paradoxical chest wall motion alone [6, 7]. 

However, that expansion has not been uniformly confirmed. In a randomized trial of patients with severe chest wall injury, Meyer et al. did not demonstrate superiority of SSRF over nonoperative management in a broader population. This tension is important because it suggests that SSRF has developed not as a universal solution, but as a selective intervention whose success depends on phenotype, timing, technical execution, and strict indication discipline [1, 2, 35–38]. 

**Fig. 1:**
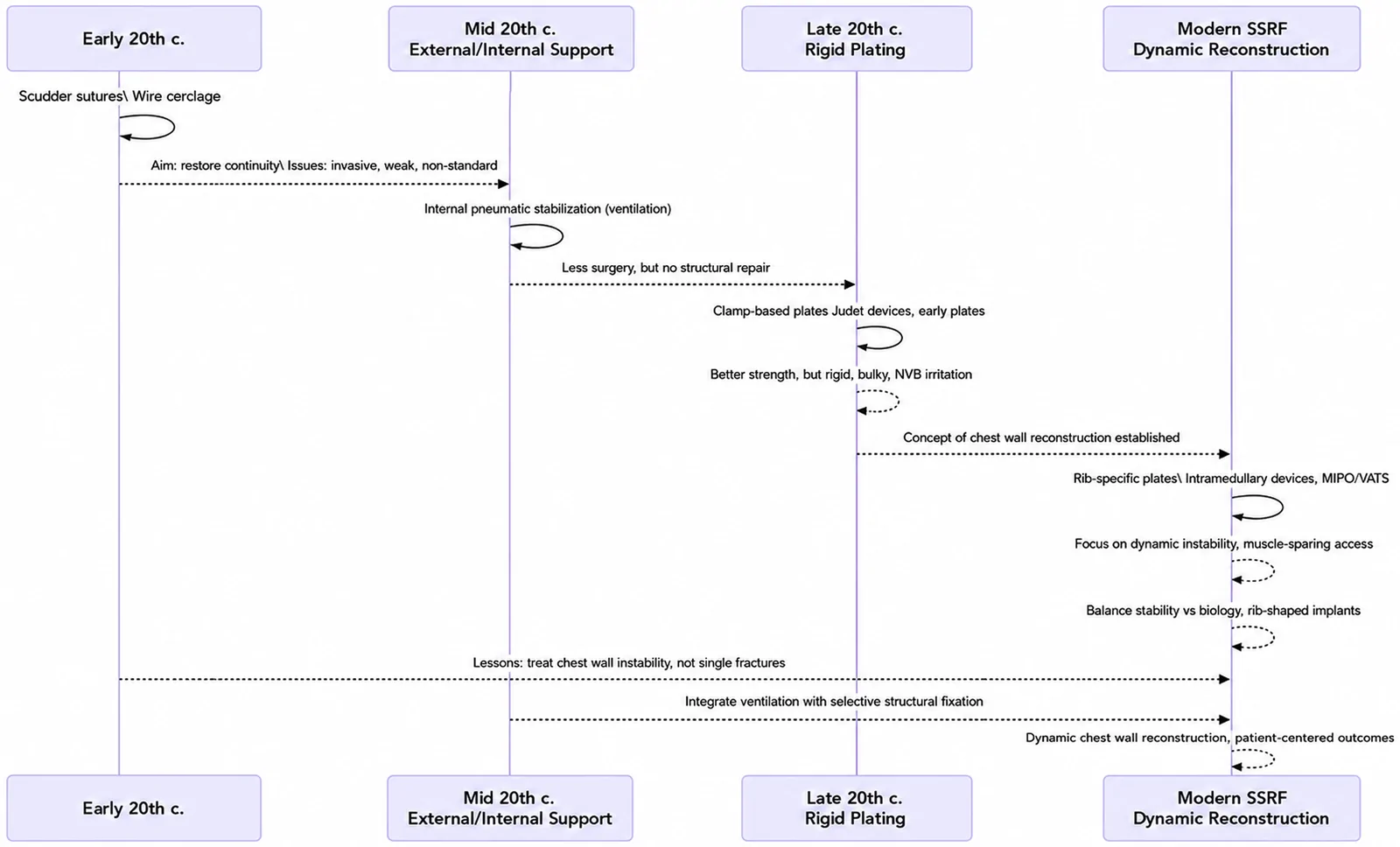
Graphical overview of the historical development of chest wall reconstruction with developing the understanding of restoring a dynamic system of breathing and functional tissue rather than just fixing fractures with implants

The historical arc is better viewed as delayed maturation than rediscovery. Rib fixation moved from plausible but weakly validated mechanical concepts to selective, evidence-supported chest wall stabilization, accompanied by a growing understanding that the thorax is a biologically and biomechanically dynamic system (Fig. 1). What changed was not the basic idea of restoring chest wall continuity, but the technical, biological, and evidentiary environment in which that idea could be applied. Modern practice benefited from the availability of rib-specific implants, more accurate imaging and diagnostic techniques, improved perioperative care, and greater respect for intact soft tissue and muscle function, all aimed at restoring effective respiratory mechanics (Fig. 1).

### Implications for current practice and future directions

The practical implication is not to make SSRF either restrictive or expansive by default. Current evidence supports fixation most strongly in flail chest and in selected non-flail patterns, but implementation data suggest that eligible patients may still be missed. In a recent single-center review, only 29.1% of patients meeting SSRF criteria actually underwent fixation, while collaborative-center data continue to demonstrate substantial variation in case volume and practice patterns [39, 40]. The task is therefore to apply accepted indications consistently through dedicated chest wall programs, with attention to timing, contraindications, fracture pattern, and local expertise. The recent WSES/CWIS position paper and the newer CWIS guidelines on indications, contraindications, and timing are especially important in this respect, because they move the field from general enthusiasm toward a more explicit decision framework [1, 2, 6, 14]. 

A second implication is that access should be discussed more carefully than simply calling for smaller incisions. The durable historical lesson is not that the shortest exposure is always best, but that the least biologically costly exposure that still permits safe reduction and mechanically sound fixation is best. In practical terms, this means prioritizing a muscle-sparing strategy when feasible, not treating incision length as a goal in itself. Some fracture patterns—especially complex posterior, upper-rib, subscapular, or multilevel segmental injuries, or cases requiring concomitant pleural evacuation or intrathoracic assessment—may justify broader exposure if that is what is required to stabilize the clinically important fractures well. Recent comparative data support better long-term lung function, shoulder motion, and thoracic motion with muscle-sparing techniques, while contemporary guidance continues to emphasize approach selection according to fracture map, chest wall anatomy, and operative objectives rather than according to a minimal-incision dogma [1, 11, 13, 14]. 

A third implication is that construct success depends not only on implant choice, but on what is actually fixed. Historical and contemporary data both suggest that in flail or segmental patterns, incomplete stabilization may leave the residual unstable component to continue moving, collapsing, or loading the construct [15–17, 41]. Marasco et al. showed that fixing only one fracture per rib in a flail segment did not prevent deformity and displacement, particularly in posterior fractures. Nickerson et al. reported better 3-month total lung capacity after complete rather than partial flail stabilization, and Perugini et al. later showed improved fracture union when both sides of flail segment rib fractures were fixed, even though short-term perioperative outcomes were similar. The practical implication is that the question is not only whether to plate, but whether the planned construct neutralizes the mechanically relevant instability. In some patients, that will require fixation of both fractures in a segmental rib, or at minimum fixation of every clinically important fracture that meaningfully contributes to the unstable chest wall pattern [14–17]. 

A fourth implication is that implant development should not be framed as a finished story of titanium plates. The historical failure modes were predictable: excessive bulk, poor conformity to rib curvature, stiffness mismatch, neurovascular irritation, loosening, migration, and hardware-related pain. From a translational standpoint, the next design target is not simply a stronger implant, but a construct that provides enough stability for healing while reducing prominence, preserving respiratory mechanics, limiting intercostal irritation, and allowing reliable postoperative assessment. Titanium remains the current standard and has solved many problems of earlier fixation systems. PEEK is relevant because it is intended to address some of the remaining concerns, including stiffness mismatch, implant prominence, hardware tolerance, imaging artefact, and better biocompatibility. Early multicenter pilot data are encouraging, with low complication rates, no hardware failure in the assessed cohort, favorable patient-reported outcomes, and 80% complete union among the PEEK-plated fractures evaluated by 6-month CT [19]. However, these data remain early, descriptive, and insufficient for strong comparative claims. PEEK should therefore be presented as a promising biomaterial platform, not as a proven replacement for established titanium systems, and larger comparative studies with longer follow-up will be required before any firm conclusions can be drawn [2, 19]. 

Future work should be more granular. The field needs to define which phenotypes benefit, how timing interacts with injury pattern, which fractures must be fixed for a construct to be mechanically complete, how exposure can be reduced without compromising reduction, how new materials behave over time, and which long-term outcomes actually matter to patients. For implant studies specifically, future comparative trials should report not only union and hardware failure, but also hardware-related symptoms, chronic pain, implant removal, respiratory function, shoulder-girdle motion, thoracic motion, return to work, imaging interpretability, and patient-reported quality of life. This agenda is justified by the current evidence landscape: prospective non-flail data support benefit in carefully selected patients, but randomized evidence in broader severe chest wall injury populations remains mixed, while recent meta-analytic work still identifies low-certainty evidence and the need for better targeted trials. Just as importantly, the CWIS has now issued recommendations for long-term follow-up, which underscores that future studies should not stop at ventilator days and pneumonia, but should systematically capture union, chronic pain, hardware symptoms, respiratory function, shoulder function, return to work, and reintervention. The next phase of SSRF should be defined by better phenotyping, better execution, and better implementation—not by another broad argument over whether fixation is good or bad [6, 14, 32, 36, 42]. 

Historically, most rib fixation reports concentrated on perioperative endpoints such as ventilator days, pneumonia, and hospital length of stay, reflecting the dominant concerns of earlier eras and the limited availability of validated long-term outcome measures. The contemporary shift toward SSRF as part of dynamic chest wall reconstruction logically extends this arc: as constructs and indications evolve, so too must the outcomes by which they are judged. Patient-reported outcomes are particularly important in chest wall injury because the clinically relevant burden often extends well beyond conventional perioperative endpoints. Persistent pain, reduced work capacity, dyspnea, hardware-related symptoms, and psychological or socioeconomic limitations may remain long after pneumonia, ventilator days, or hospital length of stay have resolved. Future SSRF studies should therefore distinguish radiographic or construct-related success from patient-centered recovery and avoid treating all chest wall injury patients as a homogeneous outcome population; pain-dominant, mechanical-instability-dominant, pulmonary-function-limited, hardware-symptom-dominant, and work-capacity-limited phenotypes may follow different trajectories. Earlier rib fracture outcome literature was constrained by the absence of validated rib- or chest-wall-specific PROMs, but newer work—including the C-QoL instrument and structured work-productivity assessment—now offers a more appropriate framework for evaluating recovery from the patient’s perspective [43–47]. 

## Limitations

This review has several limitations. First, it was designed as a literature, problem-oriented historical review rather than a systematic review and thus does not claim exhaustive literature retrieval. Second, the underlying historical literature is heterogeneous and includes technical reports, case-based descriptions, and low-level evidence, which limits direct comparison across eras. Third, some older fixation concepts are known primarily through historical reviews and selective technical reports rather than robust primary outcome studies. However, the purpose of this review was not quantitative synthesis, but conceptual interpretation of how earlier fixation strategies addressed recurring mechanical and operative problems that remain relevant to modern SSRF.

Fourth, the problem-oriented synthesis inevitably reflects the authors’ interpretation of how historical techniques map onto contemporary SSRF practice; other experts might emphasize different aspects of the same literature. These limitations mean that the present review should be read as a conceptual and hypothesis-generating framework rather than as definitive guidance.

## Conclusion

The history of SSRF is not a catalogue of obsolete implants. It is a record of surgeons repeatedly confronting the same problem: an unstable chest wall that compromises respiratory mechanics. Earlier techniques often failed because the implants, exposure strategies, and evidence base were not yet good enough for consistent and biologically acceptable use. Modern SSRF represents the maturation of that mechanical idea into a broader strategy for treating chest wall instability. Used well, it requires clear indications, appropriate timing, anatomy-specific planning, and constructs that stabilize the fractures that actually matter. That discipline cuts both ways: avoiding indiscriminate fixation while also not missing patients who meet evidence-based indications. Reinterpreted through this lens, the history of SSRF offers a frame for better selection, better execution, and a more intelligent evolution of dynamic chest wall reconstruction.

## Supplementary Information

Below is the link to the electronic supplementary material.

Supplementary Material 1

## Data Availability

No datasets were generated or analysed during the current study.
