# Supplementary material for: From rib fixation to dynamic chest wall reconstruction: historical lessons for modern surgical stabilization of rib fractures
Source: Eur J Trauma Emerg Surg. 2026 Sep 4;52(1):284. doi: 10.1007/s00068-026-03324-z (PMC13545103; doi:10.1007/s00068-026-03324-z)
Supplement: Supplementary file 1 [file 68_2026_3324_MOESM1_ESM.docx]

# Supplementary Table

**Supplementary Table. Key articles included in this literature review and rationale for inclusion**

This table summarizes the principal historical and contemporary references used to construct the problem-oriented framework of the review.

| **Ref.** | **Reference** | **Article type** | **Theme / domain** | **Main contribution to the review** |
| --- | --- | --- | --- | --- |
| 1 | Sermonesi G, Bertelli R, Pieracci FM, et al. Surgical stabilization of rib fractures (SSRF): the WSES and CWIS position paper. World J Emerg Surg. Oct 18 2024;19(1):33. doi:10.1186/s13017-024-00559-2 | Guideline / position paper | Current consensus | Defines current indications, timing, technique, and operative planning; anchors the historical lessons to present-day SSRF practice. |
| 2 | Franssen AJPM, Daemen JHT, Luyten JA, et al. Treatment of traumatic rib fractures: an overview of current evidence and future perspectives. J Thorac Dis. Aug 31 2024;16(8):5399-5408. doi:10.21037/jtd-23-1832 | Literature review | Current evidence overview | Provides a contemporary synthesis of indications, evidence gaps, and future directions, linking historical concepts to current chest wall care. |
| 3 | Sarani B, Pieracci FM. Contemporary management of patients with multiple rib fractures: What you need to know. J Trauma Acute Care Surg. 2024;97(3):337-342. doi:10.1097/TA.0000000000004338 | Literature review | Contemporary management / triage / SSRF indications | Provides a concise contemporary framework for multiple rib fracture care, including triage, nonoperative treatment, analgesia, SSRF indications, timing, and operative considerations; supports the framing of SSRF as part of structured chest wall trauma management. |
| 4 | Tanaka H, Yukioka T, Yamaguti Y, et al. Surgical stabilization of internal pneumatic stabilization? A prospective randomized study of management of severe flail chest patients. J Trauma. Apr 2002;52(4):727-32; discussion 732. doi:10.1097/00005373-200204000-00020 | Randomized trial | Flail chest evidence | Landmark trial supporting the modern clinical legitimacy of SSRF in severe flail chest. |
| 5 | Marasco SF, Davies AR, Cooper J, et al. Prospective randomized controlled trial of operative rib fixation in traumatic flail chest. J Am Coll Surg. May 2013;216(5):924-32. doi:10.1016/j.jamcollsurg.2012.12.024 | Randomized trial | Flail chest evidence | Prospective trial showing outcome benefit with operative fixation and reinforcing the evidence-based revival of SSRF. |
| 6 | Pieracci FM, Leasia K, Bauman Z, et al. A multicenter, prospective, controlled clinical trial of surgical stabilization of rib fractures in patients with severe, nonflail fracture patterns (Chest Wall Injury Society NONFLAIL). J Trauma Acute Care Surg. Feb 2020;88(2):249-257. doi:10.1097/TA.0000000000002559 | Prospective multicenter trial | Non-flail SSRF | Key study supporting selective expansion of SSRF beyond classic flail chest. |
| 7 | Pieracci FM, Lin Y, Rodil M, et al. A prospective, controlled clinical evaluation of surgical stabilization of severe rib fractures. J Trauma Acute Care Surg. Feb 2016;80(2):187-94. doi:10.1097/TA.0000000000000925 | Prospective controlled study | Modern clinical evidence | Supports the modern clinical role of SSRF in severe rib fracture patterns. |
| 8 | Bemelman M, Poeze M, Blokhuis TJ, Leenen LP. Historic overview of treatment techniques for rib fractures and flail chest. Eur J Trauma Emerg Surg. Oct 2010;36(5):407-15. doi:10.1007/s00068-010-0046-5 | Historical review | Historical synthesis | Core historical framework for early fixation concepts, internal versus external stabilization, and reasons for the decline of operative fixation. |
| 9 | Shaban Y, Frank M, Schubl S, et al. The History of Surgical Stabilization of Rib Fractures (SSRF). Surg Pract Sci. Sep 2022;10:100084. doi:10.1016/j.sipas.2022.100084 | Historical literature review | Historical synthesis | Main comparator review; summarizes the historical timeline that the present manuscript reinterprets through a problem-oriented lens. |
| 10 | Scudder CL. The Treatment of Fractures. WB Saunders; 1902. | Historical book | Earliest fixation era | Earliest cited source describing operative rib fixation by primary suture, establishing the historical starting point for mechanical rib stabilization. |
| 11 | Fokin AA, Hus N, Wycech J, Rodriguez E, Puente I. Surgical Stabilization of Rib Fractures: Indications, Techniques, and Pitfalls. JBJS Essent Surg Tech. 2020 Apr-Jun 2020;10(2):e0032. doi:10.2106/JBJS.ST.19.00032 | Technical review | Technique and pitfalls | Detailed modern operative reference for exposure, implant choice, and technical pitfalls. |
| 12 | Prins JTH, Van Wijck SFM, Leeflang SA, et al. Biomechanical characteristics of rib fracture fixation systems. Clin Biomech (Bristol). Feb 2023;102:105870. doi:10.1016/j.clinbiomech.2023.105870 | Biomechanical study | Implant–rib mismatch / construct behavior | Provides primary biomechanical support for the argument that rib fixation must respect rib-specific mechanics rather than long-bone logic. |
| 13 | Westin E, Olsén MF, Caragounis EC. Comparison of long-term outcome between muscle sparing and non-muscle sparing surgical techniques in rib plating. Eur J Trauma Emerg Surg. May 20 2025;51(1):212. doi:10.1007/s00068-025-02881-z | Comparative long-term study | Muscle-sparing exposure | Supports preference for muscle-sparing exposure and informs the discussion of biologically efficient surgical access. |
| 14 | Bauman ZM, Tian Y, Doben AR, et al. Chest Wall Injury Society guidelines for surgical stabilization of rib fractures: Indications, contraindications, and timing. J Trauma Acute Care Surg. 2025 Oct 1;99(4):522-532. doi:10.1097/TA.0000000000004750 | Guideline | Indications / contraindications / timing | Provides the current CWIS decision frame and anchors the discussion of disciplined indication expansion, contraindications, and timing. |
| 15 | Marasco S, Liew S, Edwards E, Varma D, Summerhayes R. Analysis of bone healing in flail chest injury: do we need to fix both fractures per rib? J Trauma Acute Care Surg. Sep 2014;77(3):452-8. doi:10.1097/TA.0000000000000375 | Clinical healing study | Flail construct completeness | Shows that incomplete fixation may leave residual instability and deformity, supporting fixation of mechanically relevant fractures. |
| 16 | Nickerson TP, Thiels CA, Kim BD, Zielinski MD, Jenkins DH, Schiller HJ. Outcomes of Complete Versus Partial Surgical Stabilization of Flail Chest. World J Surg. Jan 2016;40(1):236-41. doi:10.1007/s00268-015-3169-3 | Comparative outcomes study | Complete vs partial stabilization | Supports the concept that construct adequacy depends on what is fixed, not only on whether hardware is placed. |
| 17 | Perugini A, Iandoli J, Pelz N, et al. Is fixation of a single end of flail segment rib fractures enough? Trauma Surg Acute Care Open. 2025;10(2):e001707. doi:10.1136/tsaco-2024-001707 | Cohort study | Complete vs partial stabilization | Further supports fixation of both ends in selected segmental ribs to improve union and complete mechanical stabilization. |
| 18 | Liebsch C, Spering C, Wilke HJ. [Biomechanics of thoracic wall instability]. Unfallchirurgie (Heidelb). Mar 2024;127(3):180-187. doi:10.1007/s00113-023-01389-8 | Biomechanical review | Thoracic wall biomechanics / dynamic instability | Provides contemporary biomechanical support for interpreting severe rib fracture patterns as dynamic chest wall instability rather than static osseous discontinuity alone. |
| 19 | Nirula R, Diaz JJ, Trunkey DD, Mayberry JC. Rib fracture repair: indications, technical issues, and future directions. World J Surg. Jan 2009;33(1):14-22. doi:10.1007/s00268-008-9770-y | Technical review | Technique and indications | Early modern review connecting historical fixation principles with contemporary technical questions and indications. |
| 20 | Wang J, Sun Z, Liu Y, et al. Clinical effect of the internal fixation for rib fracture with single utility port complete video-assisted thoracoscopic surgery. J Cardiothorac Surg. 2024 Feb 5;19(1):59. doi:10.1186/s13019-024-02517-0 | Clinical study | Thoracoscopic / minimally invasive SSRF | Provides contemporary support for thoracoscopic-assisted fixation as an access-sparing evolution of earlier limited-exposure concepts. |
| 21 | Kayata H, Usui A, Terakawa K, et al. Feasibility and efficacy of video-assisted thoracoscopic surgery for the surgical stabilization of rib fractures: a single-center retrospective cohort study. Eur J Trauma Emerg Surg. Aug 22 2025;51(1):277. doi:10.1007/s00068-025-02955-y | Retrospective cohort study | Thoracoscopic / minimally invasive SSRF | Supports feasibility and clinical applicability of VATS-assisted SSRF in current practice. |
| 22 | Yang Z, Wen M, Kong W, Li X, Liu Z, Liu X. Complete uni-port video-assisted thoracoscopic surgery for surgical stabilization of rib fractures: a case report. J Cardiothorac Surg. Feb 06 2023;18(1):61. doi:10.1186/s13019-023-02167-8 | Case report | Thoracoscopic / minimally invasive SSRF | Illustrates the technical evolution toward increasingly less invasive fixation strategies. |
| 23 | Stefani A, Tormen F, Scamporlino A, Natali P, Cavallesco G, Morandi U. Surgical stabilization of severe flail chest with Judet and Sanchez-Lloret plates. A case report. Int J Surg Case Rep. Apr 2021;81:105805. doi:10.1016/j.ijscr.2021.105805 | Case report | Historical plate concepts | Modern case-based illustration of older clamp/plate concepts and their continuing technical relevance. |
| 24 | Engel C, Krieg JC, Madey SM, Long WB, Bottlang M. Operative chest wall fixation with osteosynthesis plates. J Trauma. Jan 2005;58(1):181-6. doi:10.1097/01.ta.0000063612.25756.60 | Case series / technical report | Early modern plate fixation | Represents a transitional step between historical clamp-based constructs and contemporary rib-specific fixation systems, illustrating early plate-and-screw osteosynthesis before mature dedicated rib implants. |
| 25 | Marasco S, Quayle M, Summerhayes R, Šutalo ID, Liovic P. An assessment of outcomes with intramedullary fixation of fractured ribs. J Cardiothorac Surg. Aug 05 2016;11(1):126. doi:10.1186/s13019-016-0510-3 | Clinical outcomes series | Intramedullary fixation | Supports the continuing relevance of intramedullary fixation in selected hard-to-access fracture patterns. |
| 26 | Spering C, Moerer O, White TW, Lehmann W. [Surgical reconstruction of chest wall instability : Indications, contraindications and timing of surgery]. Unfallchirurgie (Heidelb). Mar 2024;127(3):171-179. doi:10.1007/s00113-023-01400-2 | Evidence-based review / clinical algorithm | Chest wall instability / indications / timing | Supports the central framing of SSRF as treatment of chest wall instability and provides contemporary context for indication, contraindication, timing, operative strategy, and surgical extent. |
| 27 | Spering C, von Hammerstein-Equord A, Lehmann W, Dresing K. [Osteosynthesis of the unstable thoracic wall]. Oper Orthop Traumatol. Jun 2021;33(3):262-284. doi:10.1007/s00064-020-00688-2 | Operative technique article | Operative planning / access / implant strategy | Provides technical support for detailed preoperative planning, open or minimally invasive reduction, low-profile locking plates, intramedullary splints, and biologically cautious fixation near the pleura, lungs, and pericardium. |
| 28 | Bauman ZM, Tian Y, Puthoff G, Whitbeck SA, Gardner S, White TW. An early analysis of polyetheretherketone (PEEK) plates for the surgical stabilization of rib fractures: A pilot study. Injury. Dec 2024;55(12):111969. doi:10.1016/j.injury.2024.111969 | Pilot study | Biomaterials / future implants | Introduces PEEK fixation as a promising biomaterial platform and supports the future-directed implant discussion. |
| 29 | Sharma VJ, Summerhayes R, Wang Y, Kure C, Marasco SF. Surgical stabilisation of rib fractures: A meta-analysis of randomised controlled trials. Injury. Aug 2024;55(8):111705. doi:10.1016/j.injury.2024.111705 | Meta-analysis of RCTs | Modern evidence synthesis | High-value pooled randomized evidence supporting modern SSRF while clarifying uncertainty around some outcomes. |
| 30 | Sawyer E, Wullschleger M, Muller N, Muller M. Surgical Rib Fixation of Multiple Rib Fractures and Flail Chest: A Systematic Review and Meta-analysis. J Surg Res. Aug 2022;276:221-234. doi:10.1016/j.jss.2022.02.055 | Systematic review and meta-analysis | Modern evidence synthesis | Provides pooled evidence across flail and multiple-rib-fracture populations and helps define heterogeneity of benefit. |
| 31 | Beks RB, Peek J, de Jong MB, et al. Fixation of flail chest or multiple rib fractures: current evidence and how to proceed. A systematic review and meta-analysis. Eur J Trauma Emerg Surg. Aug 2019;45(4):631-644. doi:10.1007/s00068-018-1020-x | Systematic review and meta-analysis | Modern evidence synthesis | Summarizes the evidence base and highlights ongoing uncertainty, especially outside classic flail chest. |
| 32 | Hisamune R, Kobayashi M, Nakasato K, et al. A meta-analysis and trial sequential analysis of randomised controlled trials comparing nonoperative and operative management of chest trauma with multiple rib fractures. World J Emerg Surg. Mar 19 2024;19(1):11. doi:10.1186/s13017-024-00540-z | Meta-analysis / trial sequential analysis | Modern evidence synthesis | Key contemporary evidence source for effect estimates and certainty of evidence. |
| 33 | Simmonds A, Smolen J, Ciurash M, et al. Early surgical stabilization of rib fractures for flail chest is associated with improved patient outcomes: An ACS-TQIP review. J Trauma Acute Care Surg. Apr 01 2023;94(4):532-537. doi:10.1097/TA.0000000000003809 | Registry analysis | Timing / current outcomes | Suggests better outcomes with early SSRF in flail chest and strengthens the modern evidence base. |
| 34 | Hylands M, Gomez D, Tillmann B, Haas B, Nathens A. Surgical stabilization of rib fractures for flail chest: Analysis of center-based variability in practice and outcomes. J Trauma Acute Care Surg. Jun 01 2024;96(6):882-892. doi:10.1097/TA.0000000000004254 | Registry / practice-variation study | Center effects / timing | Shows that contemporary outcomes are influenced not only by fixation, but also by practice pattern and system-level variability. |
| 35 | Fitzpatrick A, Lampridis S, Gangadharan R, Melling D, Lampridis V. Prognostic factors for outcomes following surgical stabilization of rib fractures: A review of the literature. Injury. Nov 2024;55(11):111778. doi:10.1016/j.injury.2024.111778 | Literature review | Prognostic factors / heterogeneity | Supports the review’s emphasis on phenotype, selection, and the need for more precise indications. |
| 36 | Meyer DE, Harvin JA, Vincent L, et al. Randomized Controlled Trial of Surgical Rib Fixation to Nonoperative Management in Severe Chest Wall Injury. Ann Surg. Sep 01 2023;278(3):357-365. doi:10.1097/SLA.0000000000005950 | Randomized trial | Non-flail / mixed severe chest wall injury | Important counterbalance study showing that benefit is not universal and indication discipline is essential. |
| 37 | Dehghan N, Nauth A, Schemitsch E, et al. Operative vs Nonoperative Treatment of Acute Unstable Chest Wall Injuries: A Randomized Clinical Trial. JAMA Surg. Nov 01 2022;157(11):983-990. doi:10.1001/jamasurg.2022.4299 | Randomized clinical trial | Unstable chest wall injury | Strengthens the non-flail evidence discussion and underscores the importance of careful patient selection. |
| 38 | Marasco SF, Balogh ZJ, Wullschleger ME, et al. Rib fixation in non-ventilator-dependent chest wall injuries: A prospective randomized trial. J Trauma Acute Care Surg. Jun 01 2022;92(6):1047-1053. doi:10.1097/TA.0000000000003549 | Prospective randomized trial | Non-ventilated / non-flail patterns | Adds nuance to the evidence on selective benefit outside ventilated flail chest populations. |
| 39 | Bauman ZM, Khan H, Phillips J, et al. Comparison of patients who meet criteria for surgical stabilization of rib fractures versus those who actually get rib fixation: A single center review. Injury. Dec 2024;55(12):111930. doi:10.1016/j.injury.2024.111930 | Single-center retrospective review | Implementation / underuse | Documents the gap between eligibility and actual fixation, supporting the argument for structured implementation of evidence-based indications. |
| 40 | Eriksson EA, Wijffels MME, Kaye A, et al. Incidence of surgical rib fixation at chest wall injury society collaborative centers and a guide for expected number of cases (CWIS-CC1). Eur J Trauma Emerg Surg. Apr 2024;50(2):417-423. doi:10.1007/s00068-023-02343-4 | Multicenter incidence study | Case volume / implementation | Quantifies collaborative-center SSRF case volume and contextualizes real-world adoption across dedicated chest wall programs. |
| 41 | Bauman ZM, Herrman S, Cantrell E, et al. When more is more: Utilizing finite element analysis to assess chest wall injury stability after surgical stabilization of all rib fractures versus only a portion of the rib fractures. J Trauma Acute Care Surg. Dec 01 2024;97(6):872-878. doi:10.1097/TA.0000000000004450 | Finite element analysis study | Construct completeness / residual instability | Provides biomechanical support that partial stabilization may leave residual chest wall instability and that construct success depends on which fractures are stabilized. |
| 42 | Forrester JD, Choudhry MS, Fernandez-Moure J, et al. Chest Wall Injury Society recommendations for long-term follow-up after nonoperatively and operatively managed traumatic rib and sternal fractures. J Trauma Acute Care Surg. Feb 01 2025;98(2):277-286. doi:10.1097/TA.0000000000004517 | Society recommendations | Long-term follow-up | Supports the call for standardized long-term outcome assessment beyond perioperative endpoints. |
| 43 | Choi J, Khan S, Sheira D, Hakes NA, Aboukhater L, Spain DA. Prospective study of long-term quality-of-life after rib fractures. Surgery. Jul 2022;172(1):404-409. doi:10.1016/j.surg.2021.11.026 | Prospective quality-of-life study | Long-term patient-reported outcomes | Shows that rib fracture morbidity can persist beyond the acute hospitalization, supporting the need for long-term patient-centered outcome assessment. |
| 44 | Craxford S, Deacon C, Myint Y, Ollivere B. Assessing outcome measures used after rib fracture: A COSMIN systematic review. Injury. Nov 2019;50(11):1816-1825. doi:10.1016/j.injury.2019.07.002 | COSMIN systematic review | Outcome measures / PROM methodology | Demonstrates heterogeneity and limitations in rib fracture outcome measurement, supporting the need for validated chest-wall-specific PROMs. |
| 45 | Van Wijck SFM, Ritter KA, Van Lieshout EMM, et al. Development and preliminary validation of a novel chest wall specific quality of life instrument-C-QoL. J Trauma Acute Care Surg. Feb 01 2026;100(2):261-269. doi:10.1097/TA.0000000000004837 | Instrument development / preliminary validation study | Chest-wall-specific PROM / C-QoL | Introduces and preliminarily validates a chest-wall-specific quality-of-life instrument for more appropriate patient-centered assessment after chest wall injury. |
| 46 | Blythe A, Cassidy R, Diamond O, McManus K. Return to work and activity after rib-fixation for acute chest trauma: first application of a validated patient-reported outcomes assessment tool. Eur J Cardiothorac Surg. 2024 Jun;65(6):ezae192. doi:10.1093/ejcts/ezae192 | Patient-reported outcomes study | Return to work / activity after SSRF | Supports the importance of work capacity, activity recovery, and validated patient-reported assessment after rib fixation. |
| 47 | Swenson R, Tran T, Weatherby D, Wojahn R, Vang S, Nguyen MP. One-year patient-reported outcomes following surgical stabilization of rib fractures. Eur J Orthop Surg Traumatol. Aug 21 2025;35(1):355. doi:10.1007/s00590-025-04443-w | Patient-reported outcomes study | One-year recovery after SSRF | Provides SSRF-specific long-term patient-reported outcome data, supporting the argument that perioperative endpoints alone are insufficient. |
